# Natural History of Symptoms in Patients With Esophagogastric Junction Outflow Obstruction Using Standardized Surveys

**DOI:** 10.7759/cureus.74868

**Published:** 2024-11-30

**Authors:** Blaine Prichard, Zachary Pattison, Benjamin Stern, Myunghoon Kim, Ereny Demian, Gaser Ahmed, Meeta Desai, Lan Kong, Ann Ouyang

**Affiliations:** 1 Department of Medicine, Penn State College of Medicine, Hershey, USA; 2 Department of Medicine, Division of Gastroenterology and Hepatology, Penn State College of Medicine, Hershey, USA; 3 Department of Medicine, University of New England College of Osteopathic Medicine, Biddeford, USA; 4 Department of Public Health Sciences, Division of Biostatistics and Bioinformatics, Penn State College of Medicine, Hershey, USA

**Keywords:** esophageal motility disorders, esophagogastric junction, high-resolution esophageal manometry, long-term prognosis, treatment outcome

## Abstract

Background

Our aim was to assess the clinical presentation and outcomes of patients with a manometric diagnosis of esophagogastric junction outflow obstruction (EGJOO) using standardized symptom surveys and comparison to a cohort who were referred for manometry but who had a normal study.

Methods

We followed a cohort of adult patients without a mechanical obstruction who underwent high-resolution manometry at our medical center from 9/12/19 to 10/4/21 for 16 months.

Results

Thirty-seven patients with EGJOO (age: 60.8 ± 13.3; female: 25/37) were compared to 33 patients with normal manometry (age: 57.6 ± 13.7; female: 21/33). For the untreated normal manometry group, there was a decrease in dysphagia scores at the six-month follow-up (10.8 ± 10.5 vs. 6.4 ± 10.4, P = 0.009) and a decrease in reflux scores at the 16-month follow-up (11.2 ± 3.0 vs. 7.8 ± 2.8, P = 0.042). For the untreated EGJOO group, there were no statistically significant changes in symptom scores. For both cohorts, dysphagia scores at the time of manometry had an inverse relationship with the change in dysphagia scores (EGJOO: r = -0.446, P = 0.033) (normal manometry: r = -0.464, P = 0.045).

Conclusions

Patients with EGJOO have a prognosis distinct from patients referred for manometry but who have a normal study and are likely to improve. However, even in patients with EGJOO, severe symptoms are likely to improve. Further investigation of therapies is warranted.

## Introduction

Esophagogastric junction outflow obstruction (EGJOO) is an esophageal motility disorder defined on high-resolution manometry as impaired relaxation of the esophagogastric junction but with intact peristalsis [1]. It is a common diagnosis with 5-17% of all manometry studies receiving an EGJOO diagnosis [2-7].

EGJOO is also a relatively new diagnosis with the earliest characterizations of the disorder coming from Pandolfino et al. in 2008, where they described a group of patients who had impaired esophagogastric junction relaxation but with a normal pressurization front velocity on manometry, termed “functional obstruction” [8]. This led to the definition from the Chicago Classification (CC) v1.0 in 2009, where “functional esophagogastric junction obstruction” was defined as an elevated intrabolus pressure and increased frequency of compartmentalized swallows [9]. The first true definition of EGJOO, however, was by the CC v2.0 in 2012, defined as a mean integrated relaxation pressure ≥ 15 mmHg with intact peristalsis [10]. The CC v3.0 in 2014 modified the definition to use median instead of mean IRP and emphasized the heterogeneity of conditions potentially causing EGJOO [11]. Recently, CC v4.0 was introduced, which recommended that a mechanical obstruction be excluded prior to diagnosing any esophageal motility disorder [1]. It also recommended the use of both supine and upright swallows on manometry when diagnosing EGJOO [1].

Due to its high prevalence and unclear implications, there has been much interest in studying EGJOO. Studies have worked to clarify the symptomatology of EGJOO [2,12-14] and to further characterize the disorder on manometry [2,5,12,15]. Indeed, studies have revealed that dysphagia [2,12-14] and chest pain [2] are common presenting symptoms and that there are significant functional changes in manometry [3]. Studies have also worked to understand the natural history of EGJOO [4,7,13,16,17], identify factors that may affect its prognosis [7,13], and evaluate treatment options for those affected [4,14,17-23]. There are studies supporting the use of Botox injection [4,14,17-19], peroral endoscopic myotomy [14,19-21], and Heller myotomy [2,19,22,23] in patients with EGJOO. There is also evidence showing that a percentage of patients with EGJOO will have spontaneous resolution of symptoms [4,7,13,16,17], although there is limited evidence on factors that identify who these patients are. Further, many of these studies have been limited in their ability to compare EGJOO to a control group and in their ability to assess patient symptoms using a standardized symptom assessment.

Therefore, the first aim of this study was to determine the clinical presentations, manometry parameters, and barium study findings that are more likely to be found in patients with an EGJOO diagnosis, based on the CC v3.0, compared to a control group made up of patients who were referred for manometry but who had a normal study, based on CC v3.0. The second aim was to assess the natural history, potential prognostic factors, and response to treatment of patients with an EGJOO diagnosis and to compare these outcomes in patients with a normal manometry using validated symptom questionnaires.

## Materials and methods

Study population

A cohort of patients who underwent high-resolution manometry at the Penn State Milton S. Hershey Medical Center in Hershey, USA, from 9/12/19 to 10/4/21 was followed for 16 months. Only patients aged 18 years or older were eligible, and only patients who were reported to have normal manometry or EGJOO based on CC v3.0 were eligible. CC v3.0 was used because this study was initiated before the introduction of CC v4.0. This control group of patients with normal manometry consisted of symptomatic patients referred for manometry rather than asymptomatic patients so that the clinical significance of the manometry findings could be determined. All patients completed a Brief Esophageal Dysphagia Questionnaire (BEDQ) [24] and a Gastroesophageal Reflux Disease Questionnaire (GerdQ) [25] at the time of their manometry. The BEDQ by Taft et al. is a standardized scoring system to measure dysphagia symptoms [24], while GerdQ by Jones et al. is a standardized scoring system to measure reflux symptoms [25]. A letter was mailed to patients about three months after their manometry to explain the details of the study. Patients were then telephoned at about six months after their manometry for follow-up. The three-month delay in reaching out to the patients was chosen to limit any influence of our discussion on their treatment. Patients who gave their verbal consent to the study on the phone were asked the same questions for the BEDQ and GerdQ surveys, as well as questions about changes in medications and diagnostic and therapeutic interventions. Patient data were stored on REDCap (Vanderbilt University, Nashville, TN), a secure web-based depository. The next follow-up was at 16 months, where patients were asked the same questions as before. Age and sex were compared between consented and all remaining patients for both cohorts, and follow-up times were compared between the EGJOO and normal manometry cohorts. Some patients who were outside the window of time for contact at the six-month interval responded at the 16-month interval.

Exclusion criteria

First, patients who did not complete BEDQ or the GerdQ scores at either the time of manometry or follow-up were excluded from the analysis. Second, patients were asked if they had any relevant treatments prior to or following their manometry and were excluded if they had a fundoplication. Third, patients’ medical records were reviewed and patients who had a significant hiatal hernia or stricture on imaging prior to or following their manometry were excluded. A hiatal hernia was considered significant if it was described as large, measured as at least 6 cm, or was noted to be paraesophageal. A stricture was considered significant if it was described as moderate or severe. Similarly, any patient who had a hiatal hernia repair was excluded. Lastly, data from the Prescription Drug Monitoring Program, a state-run program that tracks the dispensing of federally controlled substances, were reviewed and patients who were on an opioid medication at the time of manometry or follow-up were excluded. A patient was considered to have been on an opioid if the unit quantity of their most recent refill was a number greater than or equal to the number of days prior to the time point of interest when the prescription was filled.

Patient characteristics

Patients were asked about the type and duration of the symptoms they had been having at the time of their manometry and whether they ever had a barium swallow study (including a modified study or an upper gastrointestinal series). The duration of symptoms was categorized into less than five years, five to 10 years, and more than 10 years. Patients’ medical records were reviewed to gather baseline (at the time of manometry) BEDQ scores, GerdQ scores, manometry results, and barium swallow findings. Only the most recent barium swallow study prior to the manometry study was considered. If the most recent barium swallow was performed at an outside institution, attempts were made to obtain the record; otherwise, the most recent study from our institution was used. Comparisons of these characteristics in the cohorts with findings of normal manometry and EGJOO were made.

Natural history and potential prognostic factors

The data for patients who did not receive any new relevant treatments since their manometry were compared at the six-month and 16-month follow-ups. Specifically, the BEDQ and GerdQ scores at the time of manometry and the changes in BEDQ and GerdQ scores at six-month and 16-month follow-ups were compared between the normal manometry group and the EGJOO group. The characteristics of these patients were also compared to the change in BEDQ and GerdQ scores at the six-month follow-up for both groups.

Treatments

Patients were asked at the six-month follow-up if they had any new relevant treatments (interventions or medication changes) since their manometry. Patients who had any kind of procedural intervention (pneumatic dilation or Botox injection) were placed into one of these intervention categories even if they also had a relevant medication change during the same period. Relevant medications were deemed to be anti-reflux medications (antacids, H2 receptor antagonists, and proton pump inhibitors) and pro-motility medications (calcium channel blockers, hyoscyamine, and tricyclic antidepressants). A patient who had a relevant medication started or increased in both categories was placed into the appropriate pro-motility medication group. A patient who had an antacid started or increased and either an H2 receptor antagonist or a proton pump inhibitor started or increased were placed into one of the latter categories as appropriate. The baseline and six-month BEDQ and GerdQ scores were compared for each treatment using the average and standard deviation if appropriate. For the 16-month follow-up analysis, only previously unobserved treatments (peroral endoscopic myotomy and Heller myotomy) were considered.

Statistical analysis

Comparisons of the study group to patients not included in the study were performed using Fisher’s exact test and Mann-Whitney U-test as appropriate. Comparisons of characteristics at the time of manometry between the EGJOO and normal manometry groups used these same statistical tests. For comparisons of BEDQ and GerdQ scores at different time points but within each group, paired Mann-Whitney U-tests were used. Potential prognostic factors were analyzed by comparing the change in standardized symptom scores to characteristics at the time of manometry using Mann-Whitney U-tests and Spearman correlation. SPSS Statistics (IBM, Armonk, NY) was used to perform all statistical tests.

## Results

Study population

**Figure 1:**
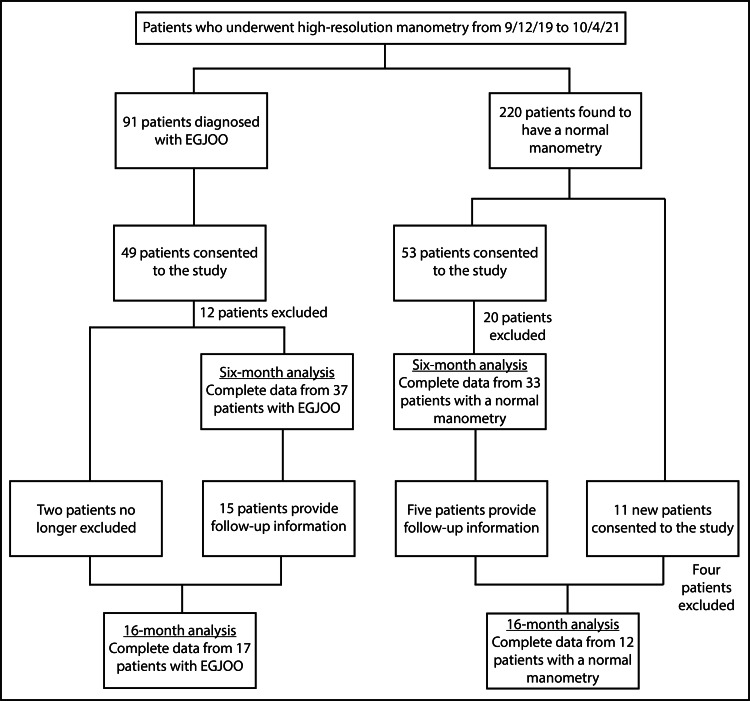
Flowchart of selection, enrollment, and exclusion of patients. EGJOO, esophagogastric junction outflow obstruction

Ninety-one patients diagnosed with EGJOO and 220 patients found to have normal manometry were eligible at the six-month follow-up (Figure 1). Of these, 49 patients with EGJOO and 53 patients with normal manometry consented to the study, but after exclusions, 37 patients with EGJOO and 33 patients with normal manometry remained. For the EGJOO cohort, exclusions were made for missing symptom surveys (one), a history of fundoplication (one), the presence of strictures (three), and opioid use (seven). For the normal manometry cohort, exclusions were made for missing symptom surveys (four), a history of fundoplication (five), a history of hiatal hernia repair (one), the presence of a large hiatal hernia (six), and opioid use (four). Most patients underwent manometry prior to the development of CC v4.0, so they only performed supine swallows; however, four patients with EGJOO underwent their studies after the introduction of CC v4.0, so they performed both supine and upright swallows, and one patient with normal manometry did likewise. The average first follow-up time was 5.9 months (3.7-9.1, SD 1.3) for patients with EGJOO and 6.1 months (4.4-10.0, SD 1.3) for patients with normal manometry. These follow-up times were not statistically different (P = 0.353).

Of the patients included in the six-month follow-up, 15 patients with EGJOO and five patients with normal manometry provided information at the 16-month follow-up. There were also new patients who provided information at the 16-month follow-up: two patients with EGJOO who had been excluded for being on a short course of opioids at the six-month follow-up - but had not been on opioids at the time of manometry - and 11 newly consented patients with a normal manometry who had been outside the six-month follow-up window before being able to contact them. After exclusions, 17 patients with EGJOO and 12 patients with normal manometry remained for the 16-month analysis. There were no exclusions in the EGJOO group. For the normal manometry group, there were exclusions for missing symptom surveys (one), a history of fundoplication (one), the presence of a hiatal hernia (one), and opioid use (one). Most patients in both groups performed only supine swallows; however, three patients with EGJOO and five patients with normal manometry performed both supine and upright swallows, and one patient in the EGJOO cohort performed only upright swallows. The average follow-up time was 16.0 months (range: 12.5-18.5, SD 1.9) for patients with EGJOO and 16.3 months (range: 13.3-19.8, SD 2.5) for patients with a normal manometry, and, again, this difference was not statistically significant (P = 0.527).

Comparison to patients not included in the study

In total, 53.8% (49/91) of patients with EGJOO and 29.1% (64/220) of patients with normal manometry consented to participate in the study. In the EGJOO group, the average age of consented patients was 61.4 (SD 12.9), and 71.4% (35/49) were female. The remainder of the eligible patients with EGJOO who were not included in the study had an average age of 59.9 (SD 12.3), and 69.0% (29/42) were female. The differences in age (P = 0.466) and sex (P = 0.822) were not statistically significant. In the normal manometry group, the average age of consented patients was 57.9 (SD 13.6), and 70.3% (45/64) were female. The remainder of the eligible patients with normal manometry had an average age of 53.8 (SD 13.0), and 76.3% (119/156) were female. While the difference between ages was statistically significant (P = 0.036), the difference between sexes was not (P = 0.395).

Patient characteristics

Characteristics were compared for the six-month follow-up cohorts (Tables 1-2). The average age of patients with EGJOO was 60.8 (SD 13.3) and more were female (25/37). The most common presenting symptoms in patients with EGJOO were dysphagia (29/37), reflux/regurgitation (13/37), and chest pain (11/37). Most patients with EGJOO had been having symptoms for less than five years (19/35). Some of the most common findings on ancillary studies in patients with EGJOO were a liquid delay on barium swallow (5/22) and a tablet delay on barium swallow (5/17). In patients with normal manometry, the average age was 57.6 (SD 13.7) and more were also female (21/33). The most common presenting symptoms in patients with normal manometry were dysphagia (22/33) and reflux/regurgitation (17/33). Unlike patients with EGJOO, most patients with normal manometry had been having symptoms for more than five years (16/29), but this finding was not statistically different between the two groups. No statistically significant differences were observed in age, sex, BEDQ score, GerdQ score, symptom profile, or barium study findings between the EGJOO and normal manometry cohorts.

**Table 1:** Quantitative characteristics of patients with esophagogastric junction outflow obstruction and normal manometry. EGJOO, esophagogastric junction outflow obstruction; BEDQ, Brief Esophageal Dysphagia Questionnaire; GerdQ, Gastroesophageal Reflux Disease Questionnaire

	EGJOO mean ± SD	EGJOO (n)	Normal manometry (mean ± SD)	Normal manometry (n)	P-value
Age	60.8 ± 13.3	37	57.6 ± 13.7	33	0.281
Percent ineffective peristalsis	16.2 ± 24.0	37	7.8 ± 11.6	33	0.346
Mean distal contractile integral (mmHg·s·cm)	3849.3 ± 3172.1	37	2006.7 ± 1054.6	33	0.027
Mean distal latency (s)	6.4 ± 1.1	37	7.2 ± 1.8	33	0.095
Mean integrated relaxation pressure (mmHg)	21.0 ± 6.2	37	7.5 ± 6.7	33	<0.001
Percent cleared bolus	53.0 ± 33.4	37	65.1 ± 28.3	32	0.117
BEDQ score	11.9 ± 10.4	37	9.2 ± 9.1	33	0.305
GerdQ score	8.3 ± 3.0	37	8.8 ± 3.2	33	0.413

**Table 2:** Qualitative characteristics of patients with esophagogastric junction outflow obstruction and normal manometry. EGJOO, esophagogastric junction outflow obstruction

	EGJOO	EGJOO (%)	Normal manometry	Normal manometry (%)	P-value
Female	25/37	67.6	21/33	63.6	0.803
Reflux/regurgitation	13/37	35.1	17/33	51.5	0.227
Chest pain	11/37	29.7	5/33	15.2	0.167
Dysphagia	29/37	78.4	22/33	66.7	0.295
Symptoms less than five years	19/35	54.3	13/29	44.8	0.616
Symptoms five to ten years	10/35	28.6	9/29	31.0	1.000
Symptoms more than ten years	6/35	17.1	7/29	24.1	0.544
Liquid delay on barium swallow	5/22	22.7	1/24	4.2	0.090
Tablet delay on barium swallow	5/17	29.4	1/11	9.1	0.355

The mean integrated relaxation pressure was elevated in patients with EGJOO as compared to patients with a normal manometry (21.0 ± 6.2 mmHg vs. 7.5 ± 6.7 mmHg, P < 0.001), as expected, given the definition of EGJOO. The mean distal contractile integral was also greater in patients with EGJOO (3849.3 ± 3172.1 mmHg·s·cm vs. 2006.7 ± 1054.6 mmHg·s·cm, P = 0.027).

Natural history

**Figure 2:**
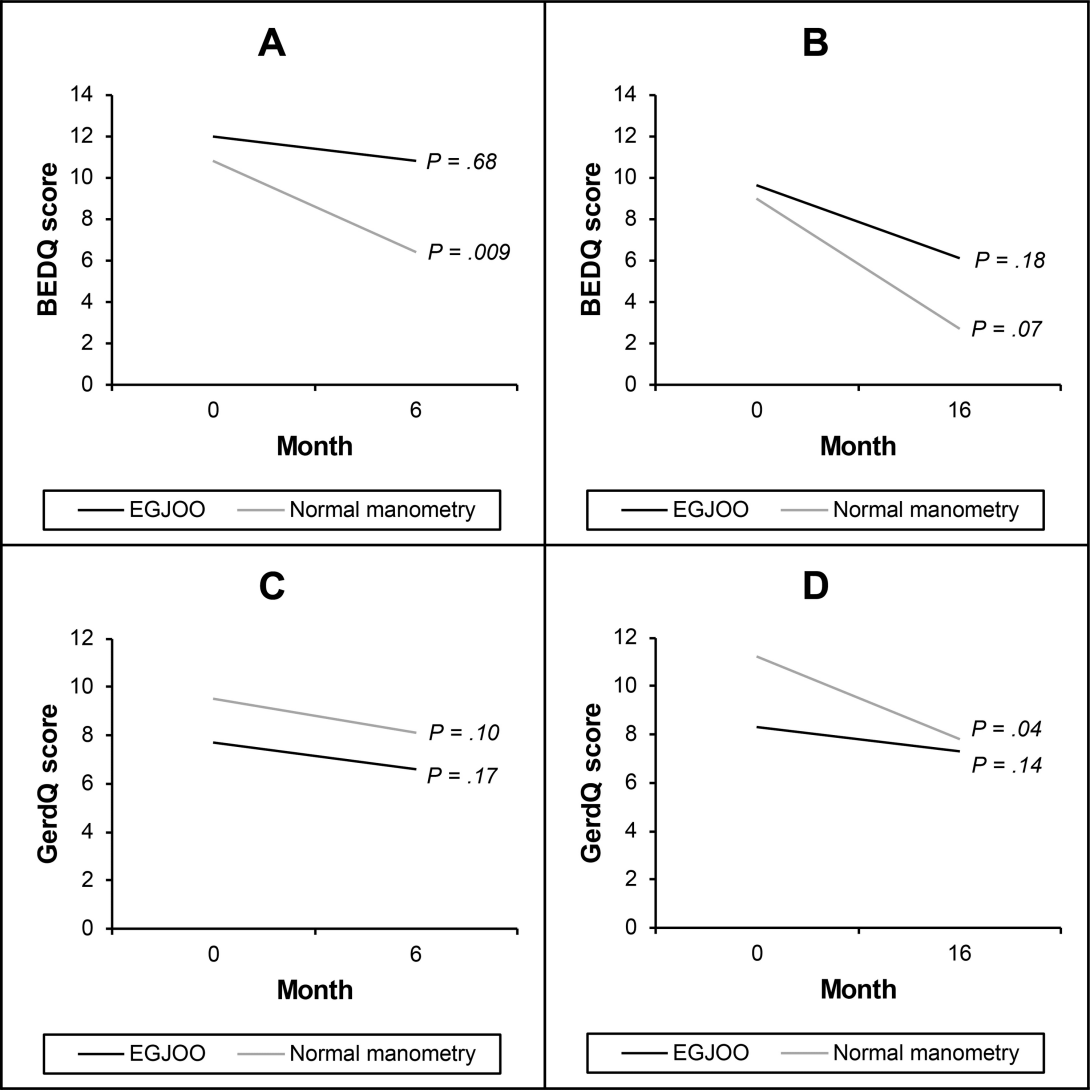
Change in mean dysphagia and reflux scores in untreated patients. Represented above are mean dysphagia scores at six (A) and 16 months (B) and reflux scores at six (C) and 16 months (D). BEDQ, Brief Esophageal Dysphagia Questionnaire; EGJOO, esophagogastric junction outflow obstruction; GerdQ, Gastroesophageal Reflux Disease Questionnaire

Over the six-month follow-up, 19 patients with normal manometry and 23 patients with EGJOO did not receive any new relevant treatments. Over the 16-month follow-up, six patients with normal manometry and nine patients with EGJOO did not receive any new relevant treatments. In the untreated normal manometry group, there was a decrease in BEDQ scores at the six-month follow-up (10.8 ± 10.5 vs. 6.4 ± 10.4, P = 0.009) and a decrease in GerdQ scores at the 16-month follow-up (11.2 ± 3.0 vs. 7.8 ± 2.8, P = 0.042) compared to baseline (Table 3 and Figure 2). For the untreated EGJOO group, there were no statistically significant differences in BEDQ or GerdQ scores at either time point.

**Table 3:** The association between baseline and the change in standardized symptom scores. The first column shows the diagnosis group, scoring system used, and follow-up time for each dataset. BEDQ, Brief Esophageal Dysphagia Questionnaire; EGJOO, esophagogastric junction outflow obstruction; GerdQ, Gastroesophageal Reflux Disease Questionnaire

	n	Baseline (mean ± SD)	Follow-up (mean ± SD)	P-value
Normal manometry, BEDQ, six-month	19	10.8 ± 10.5	6.4 ± 10.4	0.009
EGJOO, BEDQ, six-month	23	12.0 ± 10.8	10.8 ± 10.3	0.681
Normal manometry, BEDQ, 16-month	6	9.0 ± 10.5	2.7 ± 3.0	0.068
EGJOO, BEDQ, 16-month	9	9.6 ± 10.1	6.1 ± 5.0	0.181
Normal manometry, GerdQ, six-month	19	9.5 ± 3.3	8.1 ± 3.6	0.096
EGJOO, GerdQ, six-month	23	7.7 ± 2.8	6.6 ± 2.7	0.165
Normal manometry, GerdQ, 16-month	6	11.2 ± 3.0	7.8 ± 2.8	0.042
EGJOO, GerdQ score, 16-month	9	8.3 ± 2.1	7.3 ± 1.9	0.137

Prognostic factors

**Figure 3:**
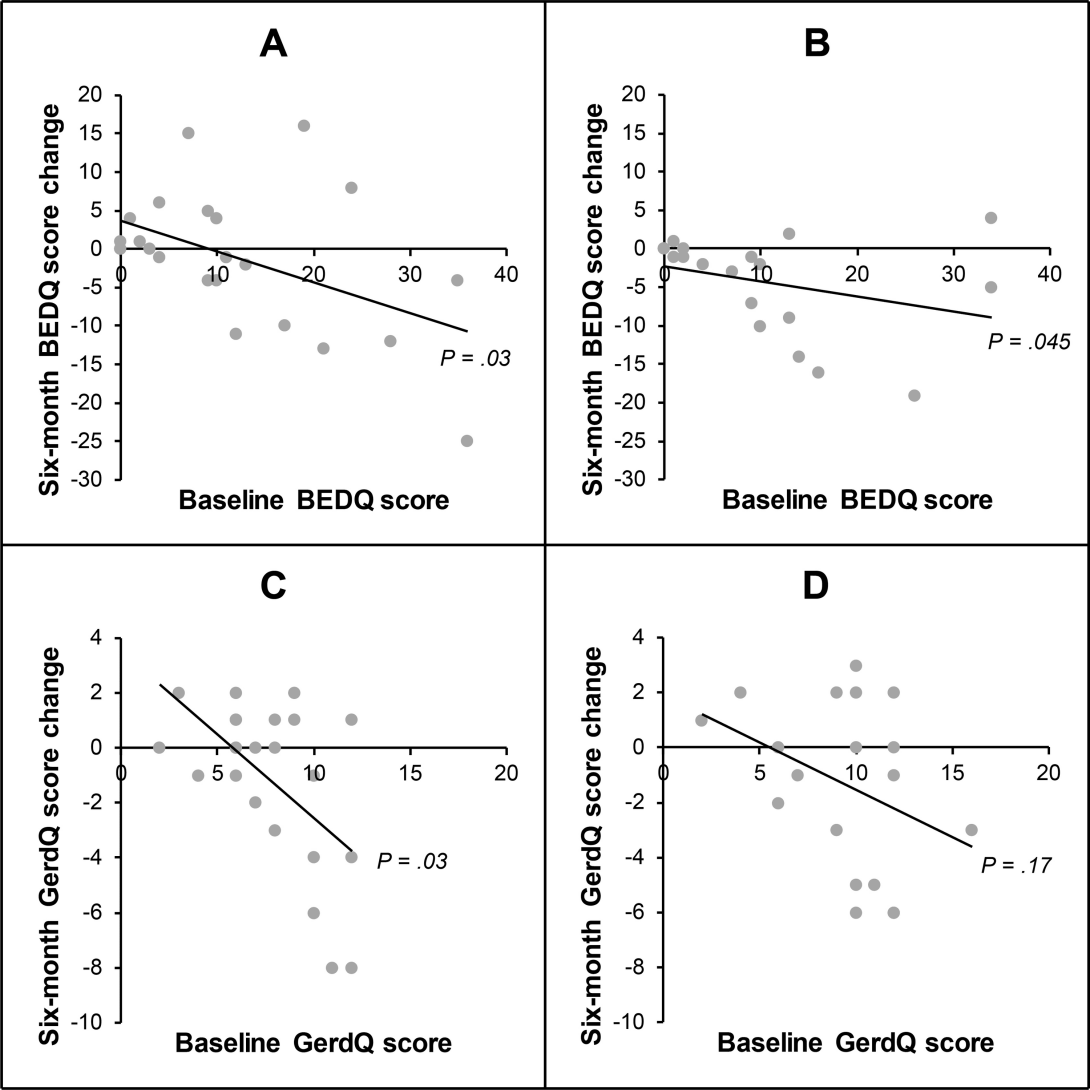
Relationship of baseline symptom scores and change in these scores in untreated patients. (A) and (C) represent patients with esophagogastric junction outflow obstruction. (B) and (D) represent patients with normal manometry. BEDQ, Brief Esophageal Dysphagia Questionnaire; GerdQ, Gastroesophageal Reflux Disease Questionnaire

**Figure 4:**
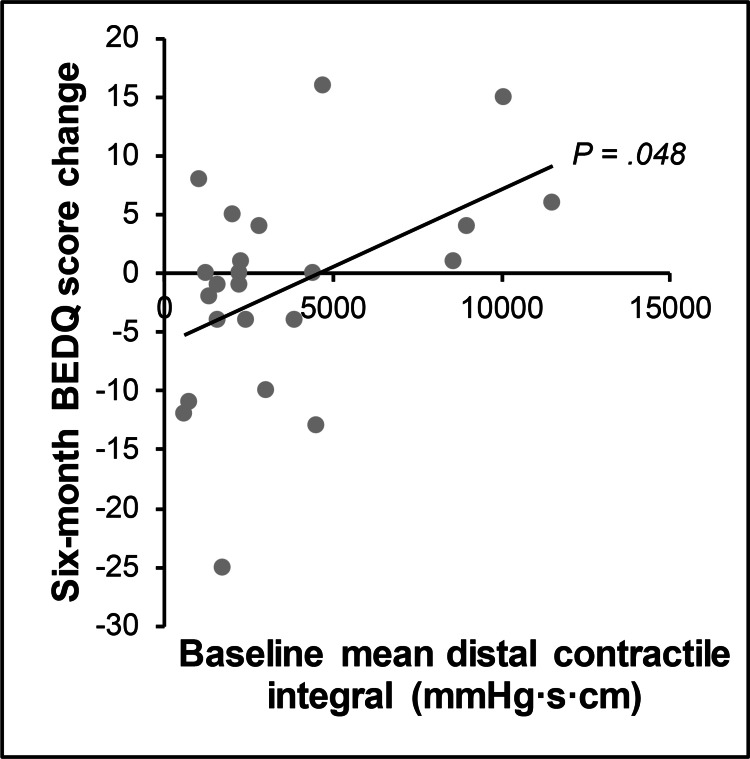
Relationship between mean distal contractile integral and change in dysphagia score. Represents patients with esophagogastric junction outflow obstruction. BEDQ, Brief Esophageal Dysphagia Questionnaire

Patient characteristics were compared with the change in BEDQ and GerdQ scores at six-month follow-up in untreated patients. Regarding symptom profiles, baseline BEDQ scores had an inverse relationship with the change in BEDQ scores in both the EGJOO (r = -0.446, P = 0.033) and normal manometry (r = -0.464, P = 0.045) cohorts (Table 4 and Figure 3). Baseline GerdQ scores had an inverse relationship with the change in GerdQ scores, but only in the EGJOO cohort (r = -0.463, P = 0.026). Baseline GerdQ scores also had an inverse relationship with the change in BEDQ scores, but only in the normal manometry cohort (r = -0.660, P = 0.002). Regarding manometry findings, there was a direct relationship between baseline mean distal contractile integral and the change in BEDQ scores for patients with EGJOO (r = 0.416, P = 0.048) (Table 5 and Figure 4). However, there was not a statistically significant relationship between mean integrated relaxation pressure and the change in BEDQ scores for patients with EGJOO (r = 0.319, P = 0.137), nor was there a statistically significant relationship between mean distal contractile integral and mean integrated relaxation pressure in these patients (r = 0.099, P = 0.652). The manometry studies for the four patients with a mean distal contractile integral greater than 8000 mmHg·s·cm were reviewed, and none of them met the criteria for concomitant diagnosis of distal esophageal spasm. There were no significant relationships between age, sex, or barium swallow study parameters and the change in BEDQ or GerdQ scores (Table 6).

**Table 4:** The association between baseline and the change in standardized symptom scores in untreated patients. The first column shows the variables used in the correlation analysis and the diagnosis group for the data set. BEDQ, Brief Esophageal Dysphagia Questionnaire; EGJOO, esophagogastric junction outflow obstruction; GerdQ, Gastroesophageal Reflux Disease Questionnaire

	r	n	P-value
Baseline BEDQ, six-month BEDQ change, EGJOO	-0.446	23	0.033
Baseline BEDQ, six-month GerdQ change, EGJOO	0.043	23	0.847
Baseline GerdQ, six-month BEDQ change, EGJOO	-0.014	23	0.951
Baseline GerdQ, six-month GerdQ change, EGJOO	-0.463	23	0.026
Baseline BEDQ, six-month BEDQ change, normal manometry	-0.464	19	0.045
Baseline BEDQ, six-month GerdQ change, normal manometry	-0.041	19	0.867
Baseline GerdQ, six-month BEDQ change, normal manometry	-0.660	19	0.002
Baseline GerdQ, six-month GerdQ change, normal manometry	-0.346	19	0.146

**Table 5:** Potential quantitative prognostic factors for patients with esophagogastric junction outflow obstruction. BEDQ, Brief Esophageal Dysphagia Questionnaire; GerdQ, Gastroesophageal Reflux Disease Questionnaire

	r		n		P-value
Age	0.218		23		0.318
Percent ineffective peristalsis	-0.098		23		0.657
Mean distal contractile integral (mmHg·s·cm)	0.416		23		0.048
Mean distal latency (s)	0.037		23		0.865
Mean integrated relaxation pressure (mmHg)	0.319		23		0.137
Percent cleared bolus	-0.144		23		0.513
Baseline BEDQ score	-0.446		23		0.033
Baseline GerdQ score	-0.014		23		0.951

**Table 6:** Potential qualitative prognostic factors for patients with esophagogastric junction outflow obstruction.

	Yes (mean ± SD)	Yes (n)	No (mean ± SD)	No (n)	P-value
Female	-2.4 ± 8.1	18	3.4 ± 11.8	5	0.403
Reflux/regurgitation	-2.0 ± 13.1	8	-0.7 ± 6.6	15	0.825
Chest pain	3.2 ± 6.7	6	-2.7 ± 9.5	17	0.256
Dysphagia	-2.3 ± 9.4	18	3.0 ± 7.3	5	0.363
Symptoms less than five years	-1.3 ± 9.5	13	-1.0 ± 9.0	10	1.000
Symptoms five to 10 years	-3.4 ± 8.6	5	-0.6 ± 9.4	18	0.587
Symptoms more than 10 years	1.4 ± 9.7	5	-1.9 ± 9.1	18	0.587
Liquid delay on barium swallow	0.5 ± 0.7	2	-1.9 ± 10.5	11	0.641
Tablet delay on barium swallow	-3.0 ± 9.5	3	-0.3 ± 3.1	7	0.833

Treatments

All relevant new treatments since manometry were considered in the six-month follow-up cohorts. These participants had been excluded from the natural history and prognostic factors analyses. In the EGJOO group, observed treatments included antacids (one), H2 receptor antagonists (two), proton pump inhibitors (three), calcium channel blockers (two), hyoscyamine (one), pneumatic dilation (three), and Botox injection (one). In the normal manometry group, observed treatments included antacids (one), H2 receptor antagonists (three), proton pump inhibitors (three), tricyclic antidepressants (three), and pneumatic dilation (one). Only previously unobserved treatment modalities were considered for the 16-month follow-up cohorts, and both were in the EGJOO cohort, which included one peroral endoscopic myotomy and one Heller myotomy. The average BEDQ and GerdQ scores at baseline and follow-up were calculated for all these treatments (Table 7).

**Table 7:** Standardized symptom scores for treatment modalities. The first column shows the treatment modality, follow-up time, and diagnosis group for the dataset. BEDQ, Brief Esophageal Dysphagia Questionnaire; GerdQ, Gastroesophageal Reflux Disease Questionnaire; EGJOO, esophagogastric junction outflow obstruction

	n	Baseline BEDQ (mean ± SD)	Follow-up BEDQ (mean ± SD)	Baseline GerdQ (mean ± SD)	Follow-up GerdQ (mean ± SD)
Antacid, six-month, EGJOO	1	10	4	16	8
H2 receptor antagonist, six-month, EGJOO	2	7.0 ± 7.1	8.0 ± 2.8	9.0 ± 0.0	9.0 ± 0.0
Proton pump inhibitor, six-month, EGJOO	4	13.8 ± 15.3	15.3 ± 18.0	7.8 ± 2.6	7.8 ± 0.5
Calcium channel blocker, six-month, EGJOO	2	17.0 ± 14.1	18.5 ± 14.8	10.5 ± 2.1	7.0 ± 2.8
Hyoscyamine, six-month, EGJOO	1	3	9	5	8
Pneumatic dilation, six-month, EGJOO	3	10.7 ± 9.3	9.3 ± 9.3	8.7 ± 4.2	10.3 ± 5.0
Botox injection, six-month, EGJOO	1	17	9	11	12
Peroral endoscopic myotomy, 16-month, EGJOO	1	8	1	12	6
Heller myotomy, 16-month, EGJOO	1	4	0	12	6
Antacid, six-month, normal manometry	1	0	0	7	6
H2 receptor antagonist, six-month, normal manometry	3	5.7 ± 2.1	9.0 ± 7.0	8.0 ± 2.0	7.0 ± 4.0
Proton pump inhibitor, six-month, normal manometry	6	9.3 ± 8.0	8.5 ± 12.0	8.2 ± 2.8	6.5 ± 3.4
Tricyclic antidepressant, six-month, normal manometry	3	7.3 ± 7.5	13.7 ± 14.2	8.0 ± 5.0	9.3 ± 3.1
Pneumatic dilation, six-month, normal manometry	1	5	4	6	6

## Discussion

Our study of a cohort of patients with EGJOO followed over 16 months using a control group and standardized symptom surveys found that EGJOO has a distinct clinical prognosis. Patients with EGJOO had more persistent symptoms over six- and 16-month follow-ups compared to patients who were also referred for manometry but who had a normal study. Both groups did show an inverse relationship between baseline symptom severity and the change in symptom severity. Similarly, both groups had limited data on treatments.

Our findings on patient characteristics revealed significant differences in manometry between the EGJOO and normal manometry cohorts. Specifically, we found that, in addition to a significantly elevated mean integrated relaxation pressure, which is the basis for the classification, patients with EGJOO had a significantly higher mean distal contractile integral, a finding that has some support in the literature [3,13]. Other manometry parameters that we evaluated for an association with EGJOO, including impaired bolus clearance and percent ineffective peristalsis, had more mixed findings in the literature [5,12,15,16,26]. Overall, our findings and those of others support the idea that patients with EGJOO have significant functional changes in manometry compared to those with normal manometry.

Besides manometry parameters, we looked at other patient characteristics but did not find any significant differences between the EGJOO and normal manometry cohorts. Specifically, we did not find significant differences in age, sex, symptom profile, or barium study findings. However, the findings in the literature, as well as in our study, suggest that dysphagia and chest pain are some of the most common presenting symptoms in patients with EGJOO [2,12-14].

We found that the natural history of EGJOO was without significant improvement in dysphagia symptoms, in contrast with the natural history of patients referred for manometry but who have a normal study, who have significant improvement in dysphagia symptoms over time despite lack of change with treatment. Ours is the only study that we are aware of that evaluates the natural history of EGJOO using standardized symptom surveys at multiple time points. It is also one of the only studies that compare the natural history of EGJOO to a control group with normal manometry. Although several prior studies show that a percentage of patients with EGJOO will have spontaneous resolution of their symptoms [4,7,13,16,17], the evidence also supports our conclusion that patients with EGJOO have a prognosis distinct from patients with a normal manometry, who are likely to improve over time [13].

We also studied possible prognostic factors that may predict this natural history. We found that symptom severity has an inverse relationship with the change in symptom scores for both patients with EGJOO and patients with normal manometry. In other words, severe symptoms are likely to moderate, regardless of diagnosis. We also found that mean distal contractile integral, a measure of the vigor of esophageal body contraction [1], has a direct relationship with the change in BEDQ score in patients with EGJOO. In other words, we found that an elevated mean distal contractile integral indicates a poor prognosis for symptom improvement in patients with EGJOO. This finding has some support in the literature [13], but further study is needed to evaluate its clinical impact. Other potential prognostic factors that we evaluated, including other symptoms and manometry parameters, had more mixed findings in the literature [7,13,16,27]. Further study of additional symptoms and manometry parameters may yield better prognostic factors, but in the meantime, the strongest trend identified is that severe symptoms are likely to moderate. This finding may indicate that patients referred for manometry experience an undulating course.

Our study had a limited number of participants who underwent treatment for EGJOO or normal manometry, and thus we were unable to compute meaningful statistics for this data. In the EGJOO group, one patient underwent Botox, one patient had a Heller myotomy, and one patient had a peroral endoscopic myotomy; all three reported improvement. The patients in either group who were treated with medication or dilation had more mixed results. The literature on this topic is mostly descriptive, but the findings agree that Botox injection, Heller myotomy, and peroral endoscopic myotomy tend to be the most successful treatments in patients with EGJOO [2,4,14,17-23,28,29]. Larger, multi-center studies are needed to assess these treatments more appropriately.

Our study has limitations. One limitation is the low response rate of eligible patients. Only those patients who answered our telephone calls and agreed to answer our survey questions were included. While it is possible the patients included in the normal manometry group are not representative of the population, there appears to be demographic equivalence between our sample and the population in the EGJOO group. It is important to note that having a sample that is older than the population in the normal manometry group would be more likely to lessen the impact of our natural history findings rather than enhance it. Another limitation is the use of hybrid cohorts that included both patients diagnosed by CC v3.0 and patients diagnosed by CC v4.0, which was introduced during the time of the data acquisition for this study. The major updates included in CC v4.0 were the exclusion of mechanical etiologies and the use of both supine and upright swallows [1]. Although less than 10% of patients at the six-month follow-up performed both supine and upright swallows, most patients still had ancillary imaging that we could use to exclude mechanical etiologies. Further, since CC v4.0 is more stringent, the use of CC v3.0 is more likely to lessen the impact of our findings rather than enhance it. Despite these limitations, our study has strengths in its use of a 16-month follow-up, standardized symptom scoring, and a control group.

## Conclusions

Patients with EGJOO have significant functional changes in manometry and have an uncertain prognosis. Meanwhile, patients referred for manometry but who have a normal study have preserved esophageal function, are likely to improve over time, and can be reassured. For patients with either EGJOO or a normal manometry, severe symptoms are likely to moderate, possibly indicating an undulating course. It may be prudent to follow these patients for a significant time period before invasive interventions. Further investigation of therapies is warranted, ideally with a large multi-center study.

## References

[1] Yadlapati R, Kahrilas PJ, Fox MR (2021). Esophageal motility disorders on high-resolution manometry: Chicago classification version 4.0©. Neurogastroenterol Motil.

[2] Scherer JR, Kwiatek MA, Soper NJ, Pandolfino JE, Kahrilas PJ (2009). Functional esophagogastric junction obstruction with intact peristalsis: a heterogeneous syndrome sometimes akin to achalasia. J Gastrointest Surg.

[3] DeLay K, Austin GL, Menard-Katcher P (2016). Anatomic abnormalities are common potential explanations of manometric esophagogastric junction outflow obstruction. Neurogastroenterol Motil.

[4] Lynch KL, Yang YX, Metz DC, Falk GW (2017). Clinical presentation and disease course of patients with esophagogastric junction outflow obstruction. Dis Esophagus.

[5] Zheng E, Gideon RM, Sloan J, Katz PO (2017). Esophagogastric junction outflow obstruction is often associated with coexistent abnormal esophageal body motility and abnormal bolus transit. Dis Esophagus.

[6] Ong AM, Namasivayam V, Wang YT (2018). Evaluation of symptomatic esophagogastric junction outflow obstruction. J Gastroenterol Hepatol.

[7] Chen S, Liang M, Tan N (2021). Upright integrated relaxation pressure predicts symptom outcome for esophagogastric junction outflow obstruction. J Neurogastroenterol Motil.

[8] Pandolfino JE, Ghosh SK, Rice J, Clarke JO, Kwiatek MA, Kahrilas PJ (2008). Classifying esophageal motility by pressure topography characteristics: a study of 400 patients and 75 controls. Am J Gastroenterol.

[9] Pandolfino JE, Fox MR, Bredenoord AJ, Kahrilas PJ (2009). High-resolution manometry in clinical practice: utilizing pressure topography to classify oesophageal motility abnormalities. Neurogastroenterol Motil.

[10] Bredenoord AJ, Fox M, Kahrilas PJ, Pandolfino JE, Schwizer W, Smout AJ (2012). Chicago classification criteria of esophageal motility disorders defined in high resolution esophageal pressure topography. Neurogastroenterol Motil.

[11] Kahrilas PJ, Bredenoord AJ, Fox M, Gyawali CP, Roman S, Smout AJ, Pandolfino JE (2015). The Chicago classification of esophageal motility disorders, v3.0. Neurogastroenterol Motil.

[12] Liu ZJ, Wang K, Duan LP, Xia ZW, Xu ZJ, Ge Y, Bao WH (2016). [Comparison of clinical features and high-resolution esophageal motility characteristics between esophagogastric junction outflow obstruction and type Ⅱ achalasia patients]. Zhonghua Yi Xue Za Zhi.

[13] Schupack D, Katzka DA, Geno DM, Ravi K (2017). The clinical significance of esophagogastric junction outflow obstruction and hypercontractile esophagus in high resolution esophageal manometry. Neurogastroenterol Motil.

[14] Okeke FC, Raja S, Lynch KL (2017). What is the clinical significance of esophagogastric junction outflow obstruction? Evaluation of 60 patients at a tertiary referral center. Neurogastroenterol Motil.

[15] Jain A, Baker JR, Rubenstein JH, Chen JW (2017). Bolus clearance in esophagogastric junction outflow obstruction is associated with strength of peristalsis. Neurogastroenterol Motil.

[16] Pérez-Fernández MT, Santander C, Marinero A, Burgos-Santamaría D, Chavarría-Herbozo C (2016). Characterization and follow-up of esophagogastric junction outflow obstruction detected by high resolution manometry. Neurogastroenterol Motil.

[17] van Hoeij FB, Smout AJ, Bredenoord AJ (2015). Characterization of idiopathic esophagogastric junction outflow obstruction. Neurogastroenterol Motil.

[18] Beveridge CA, Triggs JR, Thanawala SU, Ahuja NK, Falk GW, Benitez AJ, Lynch KL (2022). Can FLIP guide therapy in idiopathic esophagogastric junction outflow obstruction?. Dis Esophagus.

[19] Carlson DA, Schauer JM, Kou W, Kahrilas PJ, Pandolfino JE (2023). Functional Lumen Imaging Probe Panometry helps identify clinically relevant esophagogastric junction outflow obstruction per Chicago classification v4.0. Am J Gastroenterol.

[20] Ichkhanian Y, Sanaei O, Canakis A, Vosoughi K, Almazan E, Ghandour B, Khashab MA (2020). Esophageal peroral endoscopic myotomy (POEM) for treatment of esophagogastric junction outflow obstruction: results from the first prospective trial. Endosc Int Open.

[21] Jacobs CC, Perbtani Y, Yang D (2021). Per-oral endoscopic myotomy for esophagogastric junction outflow obstruction: a multicenter pilot study. Clin Gastroenterol Hepatol.

[22] Blais P, Bennett MC, Gyawali CP (2019). Upper esophageal sphincter metrics on high-resolution manometry differentiate etiologies of esophagogastric junction outflow obstruction. Neurogastroenterol Motil.

[23] Garbarino S, von Isenburg M, Fisher DA, Leiman DA (2020). Management of functional esophagogastric junction outflow obstruction: a systematic review. J Clin Gastroenterol.

[24] Taft TH, Riehl M, Sodikoff JB, Kahrilas PJ, Keefer L, Doerfler B, Pandolfino JE (2016). Development and validation of the brief esophageal dysphagia questionnaire. Neurogastroenterol Motil.

[25] Jones R, Junghard O, Dent J, Vakil N, Halling K, Wernersson B, Lind T (2009). Development of the GerdQ, a tool for the diagnosis and management of gastro-oesophageal reflux disease in primary care. Aliment Pharmacol Ther.

[26] Zizer E, Seufferlein T, Hänle MM (2017). Impaired bolus clearance in combined high-resolution esophageal manometry and impedance measurement helps to differentiate between esophagogastric junction outflow obstruction and achalasia. Z Gastroenterol.

[27] Song BG, Min YW, Lee H, Min BH, Lee JH, Rhee PL, Kim JJ (2019). Combined multichannel intraluminal impedance and high-resolution manometry improves detection of clinically relevant esophagogastric junction outflow obstruction. J Neurogastroenterol Motil.

[28] Lin KH, Lee SC, Huang TW, Huang HK (2017). Esophagogastric junction outflow obstruction-related functional chest pain treated using robotic-assisted thoracoscopic esophageal myotomy. J Thorac Dis.

[29] Pereira PF, Rosa AR, Mesquita LA (2019). Esophagogastric junction outflow obstruction successfully treated with laparoscopic Heller myotomy and Dor fundoplication: first case report in the literature. World J Gastrointest Surg.

